# Antibacterial and antioxidant bifunctional hydrogel based on hyaluronic acid complex MoS_2_–dithiothreitol nanozyme for treatment of infected wounds

**DOI:** 10.1093/rb/rbae025

**Published:** 2024-03-09

**Authors:** Yongping Lu, Weiqi Kang, Yue Yu, Ling Liang, Jinrong Li, Haiying Lu, Ping Shi, Mingfang He, Yuemin Wang, Jianshu Li, Xingyu Chen

**Affiliations:** Guangyuan Central Hospital, Guangyuan 628000, P.R. China; State Key Laboratory of Polymer Materials Engineering, College of Polymer Science and Engineering, Sichuan University, Chengdu 610065, P.R. China; Guangyuan Central Hospital, Guangyuan 628000, P.R. China; Guangyuan Central Hospital, Guangyuan 628000, P.R. China; Guangyuan Central Hospital, Guangyuan 628000, P.R. China; Guangyuan Central Hospital, Guangyuan 628000, P.R. China; Guangyuan Central Hospital, Guangyuan 628000, P.R. China; Guangyuan Central Hospital, Guangyuan 628000, P.R. China; Guangyuan Central Hospital, Guangyuan 628000, P.R. China; State Key Laboratory of Polymer Materials Engineering, College of Polymer Science and Engineering, Sichuan University, Chengdu 610065, P.R. China; Institute of Biomedical Engineering, College of Medicine, Southwest Jiaotong University, Chengdu 610031, P.R. China; State Key Laboratory of Polymer Materials Engineering, College of Polymer Science and Engineering, Sichuan University, Chengdu 610065, P.R. China; Institute of Biomedical Engineering, College of Medicine, Southwest Jiaotong University, Chengdu 610031, P.R. China

**Keywords:** hydrogel, wound repair, antibacterial, antioxidant, nanozyme, photothermal therapy

## Abstract

Wound repair is a complex physiological process that often leads to bacterial infections, which significantly threaten human health. Therefore, developing wound-healing materials that promote healing and prevent bacterial infections is crucial. In this study, the coordination interaction between sulfhydryl groups on dithiothreitol (DTT) and MoS_2_ nanosheets is investigated to synthesize a MoS_2_–DTT nanozyme with photothermal properties and an improved free-radical scavenging ability. Double-bond-modified hyaluronic acid is used as a monomer and is cross-linked with a PF127-DA agent. PHMoD is prepared in coordination with MoS_2_-DTT as the functional component. This hydrogel exhibits antioxidant and antibacterial properties, attributed to the catalytic activity of catalase-like enzymes and photothermal effects. Under the near-infrared (NIR), it exhibits potent antibacterial effects against gram-positive (*Staphylococcus aureus*) and gram-negative bacteria (*Escherichia coli*), achieving bactericidal rates of 99.76% and 99.42%, respectively. Furthermore, the hydrogel exhibits remarkable reactive oxygen species scavenging and antioxidant capabilities, effectively countering oxidative stress in L929 cells. Remarkably, in an animal model, wounds treated with the PHMoD_(2.0)_ and NIR laser heal the fastest, sealing completely within 10 days. These results indicate the unique biocompatibility and bifunctionality of the PHMoD, which make it a promising material for wound-healing applications.

## Graphical Abstract

**Figure:**
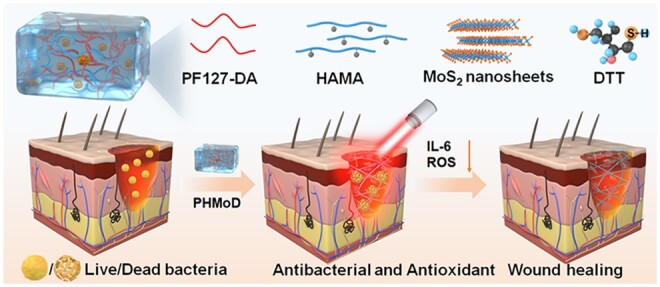

## Introduction

The skin, composed of the epidermis, dermis and subcutaneous tissue, is the body’s largest organ with protective, excretory and self-repairing functions [1–3]. External damage to the skin leads to wound formation, compromising its integrity and function [4]. Wound healing is a complex physiological process that involves several stages, such as hemostasis, inflammation, proliferation and remodelling [5]. Bacterial infection during wound repair is common and poses psychological, financial and health risks [6, 7]. Neutrophils migrate to the wound site during wound healing and release inflammatory factors contributing to inflammation. The accumulation of reactive oxygen species (ROS) can exacerbate inflammation, and uncontrolled inflammation can hasten the deterioration of wounds [8]. Hence, developing novel therapeutic approaches to address concerns such as non-healing wounds, inflammation and bacterial infections is imperative. Although antibiotics are currently the most effective and versatile treatment strategy for bacterial infections associated with wounds, prolonged use may result in bacterial resistance, leading to treatment failure [9]. Moreover, the misuse or abuse of antibiotics can lead to severe toxic side effects. The emergence of superbugs in recent years has greatly restricted the practical usage of antibiotics [10]. Therefore, developing antibacterial and anti-inflammatory therapeutics with excellent safety and efficacy options is crucial to facilitate wound healing.

Hydrogels are polymeric materials with a three-dimensional network structure formed by the chemical or physical cross-linking of polymer chains [4]. Since the discovery of hydrogel, various multifunctional hydrogels have been widely used in various fields [11]. Among them, hydrogel with antimicrobial properties have attracted increasing attention in the pharmaceutical and biomedical fields, particularly in addressing the pressing issue of bacterial drug resistance [12]. Furthermore, antimicrobial hydrogels exhibit a remarkable ability to specifically target and disrupt microorganisms’ cell membranes, employing a unique mechanism of cell lysis that significantly diminishes their likelihood of developing resistance [13]. Similarly, hydrogels have been noted for presenting excellent therapeutic results in treating inflammatory conditions. The unique three-dimensional porous structure of hydrogel materials allows them to selectively adsorb inflammatory factors, preventing excessive activation of the immune system [14]. For example, hydrogel material such as hyaluronic acid (HAMA) can promote the polarization of macrophages for inflammation suppression. As a new type of wound dressing, hydrogels possess many excellent properties, including high water contents, good hydrophilicity, biocompatibility, absorption, and anti-leakage properties, such as absorbing tissue wound exudate and maintaining a moist environment [15]. However, most current hydrogels have limitations, such as poor mechanical properties and antibacterial abilities, which make it challenging to meet the application requirements [16, 17]. In recent years, investigators have integrated several functional constituents into hydrogel systems, markedly amplifying their suitability for wound convalescence. Nanozyme, characterized by catalytic activity akin to natural enzymes, commonly exhibit a spectrum of functionalities including antimicrobial, antioxidant and photothermal attributes. The formulation of functional nanozyme-based hydrogel dressings enables the integration of diverse functions within a unified platform, thereby achieving an intelligent multifunctional therapeutic approach while upholding the inherent advantages of hydrogels. Consequently, these advancements position them as next-generation dressings designed for future applications in combating bacterial infections, mitigating unwarranted inflammation and regulating as well as fostering wound healing processes. The mechanical properties of hydrogels have been improved by introducing effective energy-dissipation mechanisms or by achieving uniform cross-linking in the hydrogel polymer network [18–20]. Furthermore, tough hydrogels can be prepared using physical association, such as micelle cross-linking, as a dynamic cross-linking method. Physical interactions are more attractive than static covalent bonds in energy dissipation because they can be easily disrupted and reorganized to produce hydrogels with considerable stretchability, self-healing and recyclability [21, 22]. Therefore, developing hydrogel with high strength and outstanding antibacterial and antioxidant ability is essential.

In recent years, nanozyme have been widely studied for excellent properties, such as catalytic efficiency, optical performance and stability. Among them, catalase-like nanozyme are a class of functional nanomaterials with similar catalytic activity to catalase [23, 24]. Molybdenum disulfide (MoS_2_), as a typical peroxidase-like nanozyme, can catalyze the breakdown of H_2_O_2_ in various substances, such as biomolecules, chemicals and environmental pollutants, to reduce the toxicity of H_2_O_2_ during wound healing. The nanozyme can degrade H_2_O_2_ to eliminate excess ROS at the wound site, thus reducing inflammation [25, 26]. Photothermal therapy (PTT) represents a treatment modality characterized by remote controllability, reduced resistance and minimal invasiveness. As such, PTT presents a potential alternative to counter the resistance issues encountered in traditional antibiotic treatments. MoS_2_ exhibits remarkable near-infrared (NIR) absorption capabilities and high photothermal conversion efficiency, rendering it an optimal candidate for employment as a photothermal agent within the NIR region [27]. Only PTT requires a localized temperature of 70°C or more to kill bacteria completely. However, too high a temperature may lead to inflammatory reactions and damage healthy tissues [28]. Therefore, introducing MoS_2_ into hydrogel for wound repair of bacterial infections while combining antioxidant properties with photothermal properties for synergistic bacterial killing can achieve complete bacterial killing at just the appropriate temperature. However, inorganic nanoparticles have disadvantages, such as high toxicity, weak molecular recognition and susceptibility to rapid removal, which limit their clinical applications [29]. To address these limitations, nanozyme hydrogel were prepared by combining hydrogel, a biocompatible polymeric material, with MoS_2_ nanosheets. Owing to the unique structural composition of nanozyme hydrogel, they possess good photothermal properties and can have a strong bactericidal effect during wound healing. Meanwhile, this reduces the risk of inorganic nanomaterials entering the human body and makes the hydrogel dual-functional as antibacterial and antioxidant agents, which is suitable for treating infected wounds [30, 31].

**Scheme 1.**
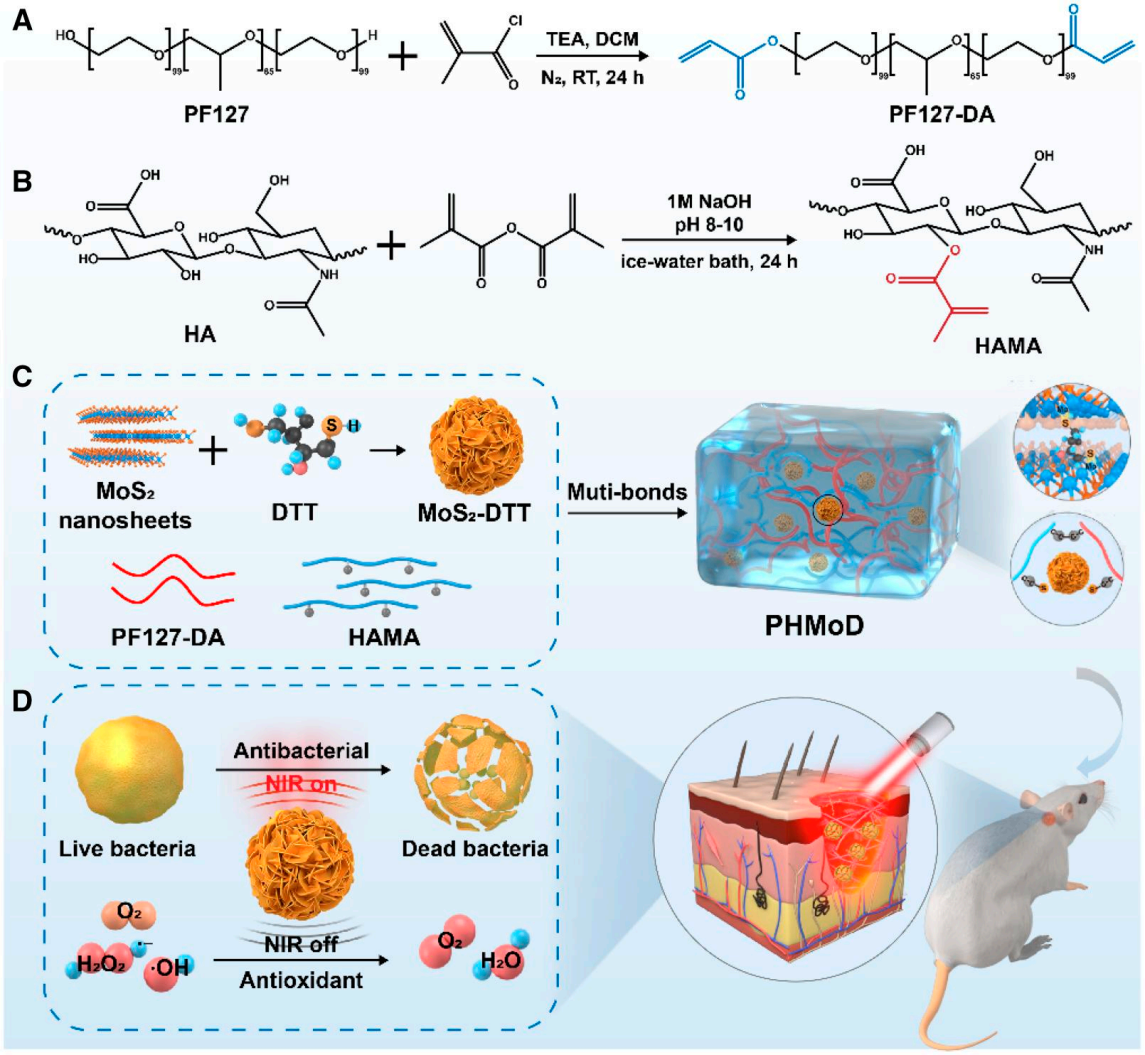
(**A**) Synthesis of PF127-DA. (**B**) Synthesis of HAMA. (**C**) Synthesis of PHMoD. (**D**) Antibacterial and antioxidant bifunctional hydrogel for treatment of infected wounds.

Herein, we prepared a hydrogel dressing with the dual function of antibacterial and antioxidant based on MoS_2_–DTT nanozyme to repair infected wounds (Scheme 1). Firstly, MoS_2_–DTT nanozyme with enhanced photothermal properties and ROS scavenging ability was prepared using the coordination between the sulfhydryl group on DTT and MoS_2_ nanosheets [32]. The cross-linker double bond grafted Planck F127 (PF127-DA) was prepared using the reaction of acryloyl chloride and PF127 in dichloromethane in the presence of triethylamine. HAMA was used as the monomer, PF127-DA as the cross-linker and MoS_2_–DTT nanozyme as the functional component copolymerized in aqueous solution to construct a bifunctional hydrogel. PHMoD have efficient antibacterial, anti-inflammatory and wound healing promotion for the treatment of infected wounds. The functional hydrogel combines the catalytic activity of catalase-like enzymes and photothermal properties with potential clinical application prospects.

## Materials and methods

### Synthesis of MoS_2_–DTT nanozyme

The following synthesis method procedure was used for preparing of MoS_2_ nanosheets was as follows [33, 34]. About 1.5 g MoS_2_ was dispersed in a mixture of 226 ml ethanol and 274 ml deionized water. The solution was then sonicated for 8 h and centrifuged at 3000 rpm for 20 min to remove any sediment. The resulting suspension was then spin-evaporated to obtain MoS_2_ nanosheets, which were dispersed in ethanol and dried in an oven.

To obtain the MoS_2_–DTT nanozyme, 20 mg MoS_2_ nanosheets and 20 mg DTT were added to 4 ml deionized water, followed by and sonication for 5 min. The resulting solution was then stirred at 50°C for 3.5 h [34].

### Synthesis of PHMoD

The synthesis method for PF127-DA has been published in previous works [33, 35]. About 2.54 g PF127 was dissolved in 20 ml dichloromethane, and 85 μl triethylamine was added. The reaction was then continued at room temperature for 24 h. The dichloromethane was removed via spinning at 25°C to obtain the crude product. The product was re-dissolved in deionized water and purified via dialysis (MWCO 3500) for 72 h. Finally, the product was lyophilized to produce both PF127 and PF127-DA [36].

To prepare the double-bond modified HAMA, 10 g HAMA (MW = 800 000–1 500 000) was added to 1 l deionized water, followed by and stirred overnight at room temperature until it was completely dissolved. After this process, 25 ml methacrylic anhydride was added dropwise to the solution in an ice-water bath, and the pH of the reaction was adjusted to 8–10 by adding 1 M NaOH. The reaction was continued for 24 h, and the pH was monitored measured at 30 min intervals and adjusted with 1 M NaOH to stabilize it. Finally, the dialysate was lyophilized to obtain HAMA [37, 38].

Through sonication, 200 mg HAMA and 400 mg PF127-DA were dispersed in 20 ml deionized water. Then, 20 mg initiator (D2959) was added to the mixture, followed by stirring and stirring until it was dissolved entirely, during which taking care to avoid light exposure to light was avoided. The resulting mixture (3.2 ml) was then added to the MoS_2_–DTT nanozyme and subjected to ultraviolet (UV) irradiation at 60°C for 15 min to obtain the PHMoD_(2.0)_ with a MoS_2_–DTT nanozyme concentration of 2.0 mg/ml. Similar methods were used to synthesize PHMoD_(1.5)_, PHMoD_(1.0)_, PHMoD_(0.5)_ and the blank hydrogel PHMoD_(0)_, all of which contained different concentrations of the MoS_2_–DTT nanozyme.

### Water retention properties and swelling properties

The prepared PHMoD_(2.0)_ hydrogel prepared according to the above method with a height of 5 mm and a diameter of 8 mm was soaked in phosphate-buffered saline (PBS) for 24 h, purified and weighed, and then placed in an oven at 37°C. The hydrogel was weighed at fixed intervals, and the cumulative water loss was calculated as follows:

(1) $$\mathrm{Water}\;\mathrm{holding}\;\mathrm{ratio}=\frac{M_{t}}{M_{s}}\times 100\%$$

*M*_t_ represents the weight of the PHMoD_(2.0)_ hydrogel at a fixed time point, and *M*_s_ represents the weight of the PHMoD_(2.0)_ after complete water absorption [38].

The PHMoD_(2.0)_ hydrogel (height of 5 mm and diameter of 8 mm) was immersed in water or wound-simulating simulated wound fluid (SWF). The mass of the hydrogel was weighed again at a fixed interval, and the swelling ratio (SR) was calculated as follows:

(2) $$\mathrm{SR}=\frac{M_{t}-M_{0}}{M_{0}}\times 100\%$$

*M*_t_ represents the weight of the PHMoD_(2.0)_ hydrogel at a fixed time point, and *M*_0_ represents the initial weight of the PHMoD_(2.0)_ hydrogel.

To prepare the SWF, 2.22 g calcium chloride, 23.38 g sodium chloride, 9.69 g Tris and 20 g bovine serum albumin were added to 1 l deionized water [39, 40].

### Photothermal properties of PHMoD

The photothermal properties of PHMoD was evaluated by means of a NIR thermography system (885-2, Testo, Germany). Firstly, the hydrogel containing different concentrations of MoS_2_ were purified by soaking them in PBS for 24 h. Then, 200 mg hydrogel was mixed with 500 μl deionized water, followed by and irradiation with an 808 nm laser (1.5 W/cm^2^) for 15 min, while the temperature value was recorded every 30 s. The effects of the laser power density (1.0, 1.5 and 2.0 W/cm^2^) and nanozyme concentration on the hydrogel warming, and the photothermal cycling stability of PHMoD_(2.0)_ was investigated [41]. The thermal images of PHMoD under 808 nm laser irradiation were also recorded directly.

### Antibacterial activity of PHMoD

*Staphylococcus aureus* (*S.aureus*) and *Escherichia coli* (*E.coli*) were selected as experimental strains for analyzing the antibacterial properties of the PHMoD. Moreover, we incubated 500 μl bacterial solutions (10^6^ CFU/ml) with 200 mg hydrogel. Without NIR irradiation group was incubated with the bacterial solution for 25 min. NIR irradiation group were incubated with *S.aureus* and *E.coli* for 10 min, then irradiated with a laser for 15 min (1.5 W/cm^2^). The solutions treated with hydrogel were diluted to 10^4^ CFU/ml. About 100 μl solution was taken added to the solid medium for plate coating experiments. The bacterial solution was incubated in a 37°C incubator for 9–12 h. The number of colonies was determined using photographs.

The bacteria were incubated in the above hydrogel via centrifugation at 3000 rpm for 5 min and then fixed in 2.5% glutaraldehyde for 4 h. Then, the bacteria were dehydrated in gradients of 25%, 50%, 75%, 90% and 100% ethanol solutions for 10 min each time. Finally, the gradient-dehydrated bacteria were dried dropwise on titanium Ti sheets in a vacuum oven at 37°C. The morphology of the bacteria after hydrogel treatment was examined using a scanning electron microscope (SU8220, Hitachi Corp, Japan).

### Antioxidant properties of PHMoD

Non-fluorescent terephthalic acid can be combined with •OH to form 2-hydroxy terephthalic acid with fluorescent properties for measuring and detecting the content of •OH. Terephthalic acid (0.5 mM), H_2_O_2_ (1.0 mM) and PHMoD (200 mg) were added to 3 ml PBS. The mixture was placed in a shaker at 37°C for 12 h, and then the fluorescence intensity of 2-hydroxyterephthalic acid was measured [42].

The hydrogel to scavenge superoxide anions was evaluated according to inhibit the photochemical reduction of nitroso blue tetrazolium (NBT) in the NADH-NBT-PMS system. First, 200 mg PHMoD was added to 16 mM Tris-HCl buffer (4.5 ml, pH 8.0), followed by the addition of 300 μM NBT (0.5 ml) and 468 μM NADH (0.5 ml). The reaction was initiated by adding 60 μM PMS (0.5 ml) to the mixture. The mixture was incubated at room temperature for 5 min, and the absorbance of the blank sample was measured at 560 nm. A decreased reduction in the absorbance of the reaction mixture indicates enhanced superoxide anion scavenging activity [43].

Intracellular ROS clearance assay was performed using L929 cells, where the ROS positive control reagent was H_2_O_2_ (final concentration was 100 μM). L929 cells (1.5 × 10^4^ cells/well) were inoculated in 48-well plates and incubated for 24 h with 10 μl PBS, 10 μl H_2_O_2_, 10 μl H_2_O_2_ + and PHMoD_(0)_, 10 μl H_2_O_2_ + PHMoD_(1.0)_ and 10 μl H_2_O_2_ + PHMoD_(2.0)_, respectively. The cells were co-cultured with a reactive oxygen fluorescent probe (DCFH-DA, 10 μM) for 20 min. After washing three times with PBS, fluorescence microscopy assessed the intracellular ROS levels by detecting the reactive oxygen fluorescent probe (λ_ex_ = 488 nm, λ_em_ = 525 nm).

The consumption of H_2_O_2_ by the hydrogel was measured using the titanium sulfate colorimetric method. After incubating 3 ml H_2_O_2_ (1 mM) and 200 mg PHMoD for 24 h at 37°C, 2 ml titanium sulfate solution was added to 1 ml supernatant. After 30 min, the UV absorption curve was recorded in the 300–600 nm. The titanium sulfate solution was prepared by dissolving 1.33 ml 24 wt% titanium sulfate solution and 8.33 ml concentrated sulfuric acid in 50 ml deionized water.

### *In vivo* wound healing

Eighteen healthy male Kunming mice (30–40 g, 8–10 weeks) were employed to evaluate the healing of bacterial infections in mice wounds promoted by PHMoD hydrogel. *In vivo* experiments were provided by the Experimental Animal Center of Sichuan University. All animal studies have animal ethics and welfare approval authorized by the Animal Ethics Committee of Sichuan University (Animal ethics number: 2019067A).

The mice were kept in polypropylene cages at room temperature (20 ± 4°C) and were provided conventional food and available drinking water. The mice were fed for 3 days to acclimate, and then their necks were shaved and anaesthetized with 1% sodium pentobarbital. The traumatized skin was disinfected with iodophor solution and 4% chlorhexidine gluconate, followed by a disposable skin biopsy perforator to create a whole skin wound with a diameter of approximately 7 mm in diameter. About 20 μl *S.aureus* solution (10^8^ CFU/ml) was added dropwise to the wounds for infection. The mice dosed with the bacterial solution were randomly divided into six groups for different treatments: control, PHMoD_(0)_, PHMoD_(2.0)_, control + NIR, PHMoD_(0)_ + NIR and PHMoD_(2.0)_ + NIR. In the NIR group, laser radiation (1.5 W/cm^2^) was used to warm the wounds to 50°C, followed by continuous irradiation for 5 min while ensuring that the real-time temperature did not exceed 55°C using a thermal imager. The wound sizes of mice were measured and photographed to document the wound repair process on 0, 3, 6, 8 and 10 days to document the wound repair processes. The hydrogel was replaced while the wounds were photographed. The measurements were performed using the obtained photographs.

### Statistical analysis

Statistical analyses were performed using the GraphPad Prism software (version 8.0). The data were expressed as mean ± standard deviation values. Data from different groups were compared via a one-way analysis of variance, and the threshold for statistical significance was set as *P *<* *0.05.

## Results and discussion

### Synthesis and characterization of PHMoD

**Figure 1.**
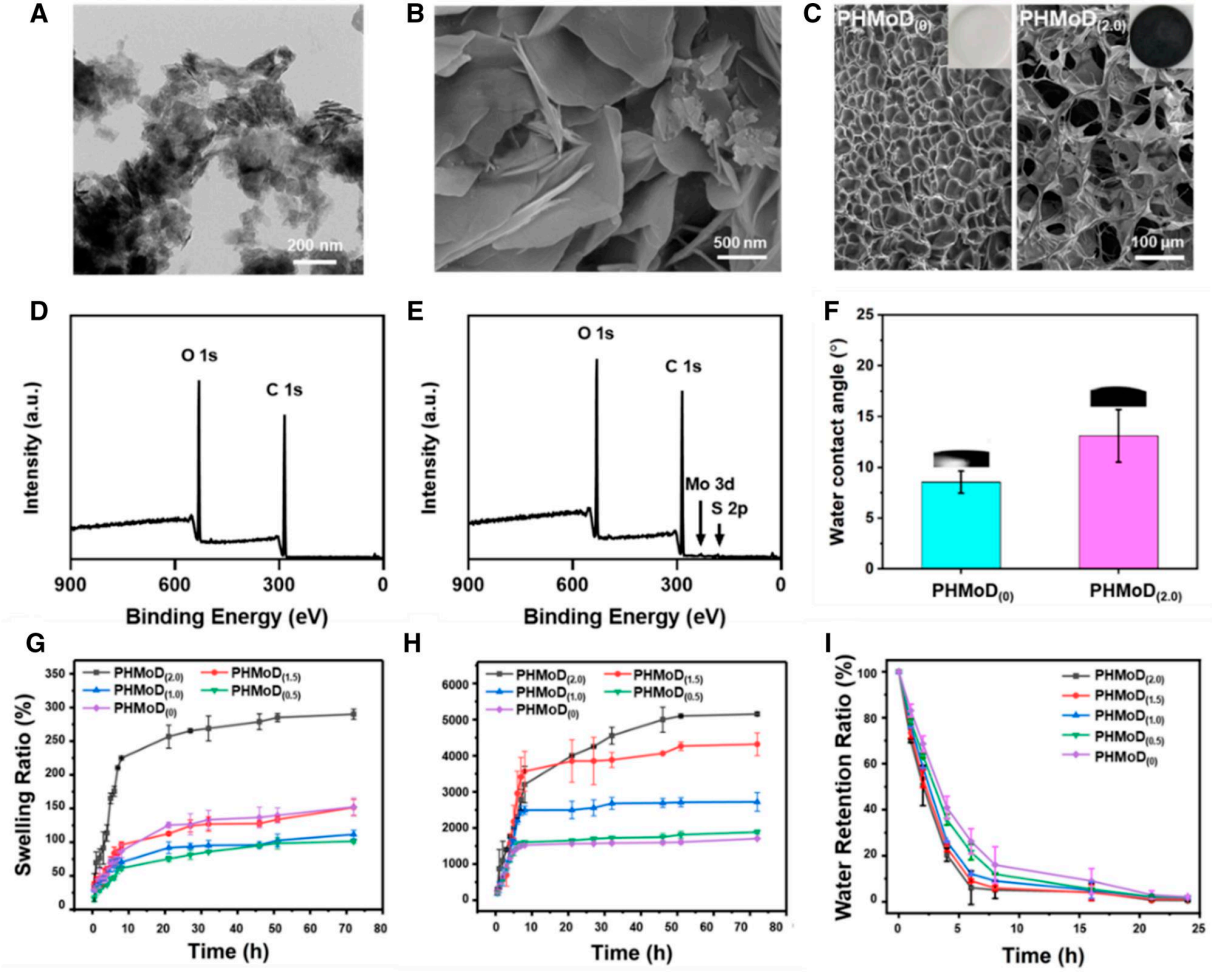
Characterization of the MoS_2_–DTT nanozyme and PHMoD. (**A**) TEM image of MoS_2_ nanosheet. (**B**) SEM image of the MoS_2_–DTT nanozyme. (**C**) Photographs and SEM images of PHMoD_(0)_ and PHMoD_(2.0)_. (**D**) XPS spectra of PHMoD_(0)_ and (**E**) PHMoD_(2.0)_. (**F**) Water contact angles of PHMoD_(0)_ and PHMoD_(2.0)_. Swelling behaviors of the PHMoD in (**G**) SWF and (**H**) water, respectively. (**I**) Water retention of the hydrogel.

MoS_2_ nanosheets were obtained via ultrasonic exfoliation. Scanning electron microscopy (SEM) and transmission electron microscopy (TEM) images of the sheet-like structure indicated that MoS_2_ had a particle size of approximately 100 nm (Supplementary Figure S1A and Figure 1A). Atomic force microscopy images confirmed that the MoS_2_ had a sheet-like structure, with a height ranging between 7 and 12 nm (Supplementary Figure S1B). The 908 cm^−1^ absorption peak appeared as an S–S bond stretching vibration absorption peak in FTIR spectrum of MoS_2_ nanosheet (Supplementary Figure S1C). In the subsequent synthesis of the MoS_2_–DTT nanozyme, the lamellar structure of MoS_2_ was maintained, as observed through SEM imaging (Figure 1B). Additionally, the C–H stretching vibration absorption peak appeared near 2900 cm^−1^. The 720 cm^−1^ absorption peak appeared as an S–C bond stretching vibration absorption peak (Supplementary Figure S2A). The results indicated the successful synthesis of the MoS_2_–DTT nanozyme. X-ray photoelectron spectroscopy (XPS) analysis confirmed the presence of Mo and S (Supplementary Figure S2B). The binding energies of the spin-split peaks for Mo were 233.2 and 229.5 eV, and those for S were 163.7 and 162.5 eV (Supplementary Figure S2C and D).

PF127-DA—a macromolecular cross-linker with functional degree 2—was prepared through the structural modification of PF127 with acryloyl chloride at the end group (Supplementary Figure S3A). The Fourier transform infrared (FTIR) spectrum of PF127-DA exhibited a new infrared absorption peak at 1724 cm^−1^ after the functional modification of PF127, which corresponded to the stretching vibration of the C = O acrylate structure (Supplementary Figure S3B). Furthermore, the proton nuclear magnetic resonance (^1^H NMR) spectra exhibited characteristic peaks of acrylate double bonds with chemical shifts of 6.47 and 5.87 ppm, which confirmed the successful synthesis of PF127-DA (Supplementary Figure S3C). Micelle formation was also detected form TEM image of PF127-DA (Supplementary Figure S3D). HAMA is abundant in reactive groups, such as hydroxyl and carboxyl groups [44]. HAMA was produced by grafting a double bond onto the HAMA unit using acrylic anhydride (Supplementary Figure S4A). The ^1^H NMR spectrum of HAMA exhibited hydrogen nucleation peaks of the acrylate double bond at 6.07 and 5.65 ppm (Supplementary Figure S4B). The FTIR spectrum of HAMA exhibited a C = O stretching vibrational absorption peak at 1725 cm^−1^, indicating the successful synthesis of HAMA (Supplementary Figure S4C).

To prepare the antioxidant and antibacterial bifunctional micellar structure of the PHMoD, the MoS_2_–DTT nanozyme as the functional component, HAMA as the monomer, and PF127-DA as a cross-linking agent was combined in an aqueous solution. Hydrogel was successfully obtained by polymerizing HAMA in an aqueous solution using PF127-DA as a cross-linking agent. Then, PHMoD hydrogel was prepared in coordination with MoS_2_-dithiothreitol (DTT) as the functional component. SEM images of the resulting PHMoD_(0)_ and PHMoD_(2.0)_ hydrogel indicated their typical three-dimensional porous structures with different pore sizes due to the addition of MoS_2_ (Figure 1C). Notably, PHMoD_(0)_ exhibited a denser cross-linked network. In contrast, the pore size of PHMoD_(2.0)_ (>50 μm) was significantly larger than that PHMoD_(0)_ (∼10 μm), indicating a dependence of the hydrogel structure formation on the MoS_2_ content. Moreover, XPS elemental analysis revealed the presence of Mo and S in PHMoD_(2.0)_ (Figure 1D and E). The addition of MoS_2_ increased the water contact angle from 8.5° for PHMoD_(0)–13_.1° for PHMoD_(2.0)_, both of which were hydrophilic (Figure 1F). Hydrophilic hydrogel creates a moist environment that promotes the healing of wounds.

The dissolution equilibrium of PHMoD_(2.0)_, PHMoD_(1.5)_, PHMoD_(1.0)_, PHMoD_(0.5)_ and PHMoD_(0)_ in SWF was reached after approximately 50 h, with dissolution rates of approximately 285.0%, 133.6%, 102.7%, 98.4% and 139.8%, respectively (Figure 1G). The SR of the PHMoD in water sharply increased during the first 10 h, with no significant increase after that except for PHMoD_(2.0)_. After 72 h, the SR of the PHMoD were 5153.3%, 4317.1%, 2721.4%, 1887.6% and 1707.1%, respectively (Figure 1H). The dissolution rate of the PHMoD in SWF was lower than that in water, likely owing to the introduction of electrolytes, which reduced the osmotic pressure difference between the gel matrix and the external solution. Furthermore, the ‘ionic cross-linking’ of the cationic hydrogel polymer reduces the osmotic pressure inside and outside the hydrogel by reducing the amount of network space within the gel, causing the hydrogel to shrink and exhibit poor swelling performance. The SR of hydrogel increased with the content of nanozyme in the hydrogel, possibly because of the presence of hydrophilic groups such as hydroxyl groups in the MoS_2_–DTT nanozyme. Additionally, the hydrogel water retention was evaluated, and it was found that the hydrogel gradually lost water in the oven at 37°C, with water contents of 0.6%, 1.0%, 1.7%, 2.0% and 2.1%, respectively, after 24 h (Figure 1I).

Under typical frequency and strain conditions, the energy storage modulus (*G*′) of the hydrogel was higher than the loss modulus (*G*″), indicating viscoelasticity. However, as the strain and frequency increased, the internal structure of the gel was disrupted and the loss modulus (*G*″) exceeded the energy storage modulus (*G*′), indicating the mobility of the PHMoD (Supplementary Figure S5). Compared with PHMoD_(0)_, PHMoD_(2.0)_ exhibited a substantially lower energy storage modulus. The inclusion of MoS_2_ hindered the formation of a uniform cross-linked structure within the hydrogel, degrading the mechanical properties of the material. Additionally, the degradation rate of PHMoD in the presence of hyaluronidase increased with the MoS_2_–DTT nanozyme concentration (Supplementary Figure S6).

### Photothermal properties of PHMoD

**Figure 2.**
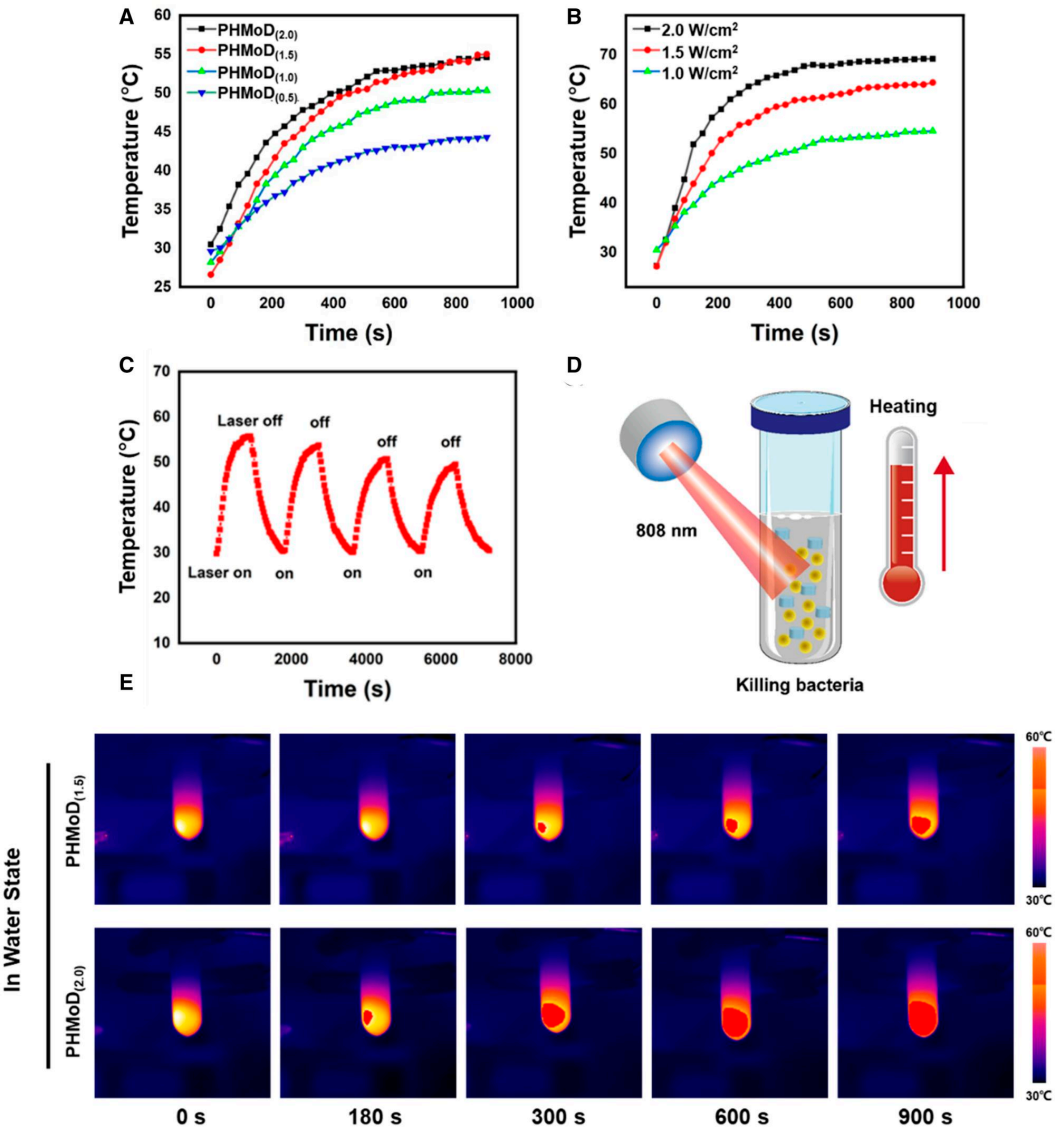
(**A**) Temperature change curves of the PHMoD with different nanozyme concentrations under NIR (1.5 W/cm^2^). (**B**) Temperature change curves of PHMoD_(2.0)_ for different power densities of the NIR laser (1.0, 1.5 and 2.0 W/cm^2^). (**C**) On–off temperature curves for PHMoD_(2.0)_ under NIR irradiation. (**D**) Photothermal process of PHMoD under NIR irradiation. (**E**) Thermal images of PHMoD_(1.5)_ and PHMoD_(2.0)_ in water under NIR irradiation (1.5 W/cm^2^).

The hydrogel was irradiated with an 808 nm NIR laser, and the temperature was measured every 30 s using thermal imaging to evaluate their photothermal efficiencies. The maximum temperature of the NIR-irradiated PHMoD increased with the MoS_2_ nanosheet concentration, and after 15 min of NIR, the temperatures of PHMoD_(2.0)_, PHMoD_(1.5)_, PHMoD_(1.0)_ and PHMoD_(0.5)_ were 54.6, 55.0, 50.3 and 44.3°C (Figure 2A). Additionally, the effects of different power levels on the photothermal performance of the hydrogel were examined. Under 15 min continuous irradiation of PHMoD_(2.0)_ with 1.0, 1.5 and 2.0 W/cm^2^ lasers, the temperature increased to 54.4, 63.8 and 69.6°C, respectively (Figure 2B). The photothermal stability of the PHMoD was examined by turning off the laser after 15 min of NIR irradiation and naturally cooling the hydrogel for 15 min at room temperature. PHMoD_(2.0)_ exhibited almost no temperature change, indicating excellent photothermal cycle stability. However, after the five cycles, the maximum temperature remained higher than 50°C, which would not significantly affect the antibacterial performance (Figure 2C). A suitable laser power is required for sterilization because a temperature that is too low cannot kill the bacteria (Figure 2D). In contrast, excessively high temperature can damage the surrounding normal tissue. Moreover, thermal images of the PHMoD (1.4 wt%, 1.5 W/cm^2^) in water were recorded using an infrared thermal imaging camera to examine the effects of the MoS_2_–DTT nanozyme content on the photothermal properties of the PHMoD. The temperature of PHMoD_(1.5)_ in water increased to 48.4°C under continuous exposure to the 808 nm NIR laser for 900 s. In contrast, the temperature of PHMoD_(2.0)_ increased to 58.3°C (Figure 2E). From the results, it can be inferred that increasing the MoS_2_–DTT nanozyme concentration can improve the photothermal properties of the PHMoD.

### Antibacterial properties of PHMoD

**Figure 3.**
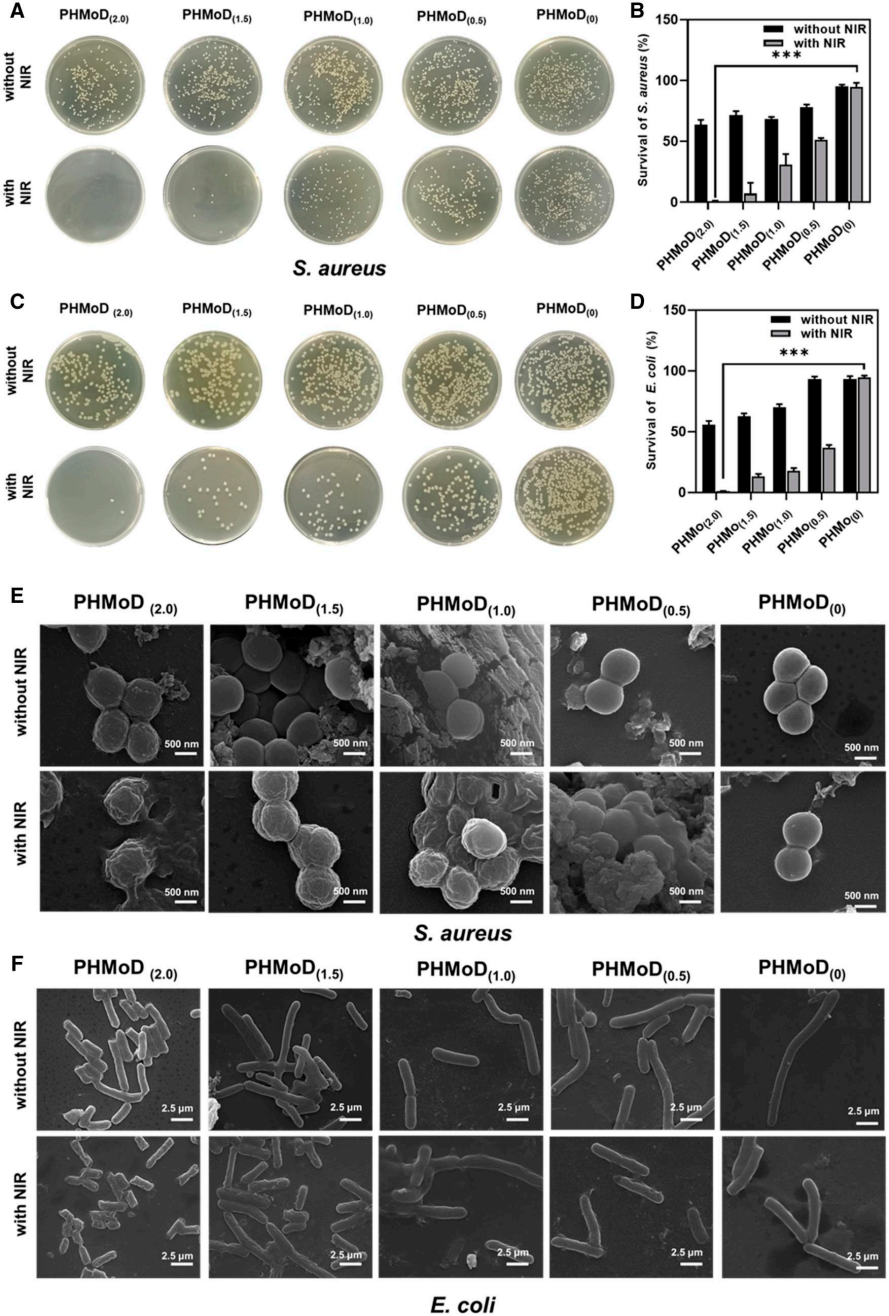
(**A**) Photographs of bacterial colonies formed by *S.aureus* and (**B**) survival rates of *S.aureus*. (**C**) Photographs of bacterial colonies formed by *E.coli* and (**D**) survival rates of *E.coli*. SEM images of (**E**) *S.aureus* and (**F**) *E.coli* after being treated by PHMoD_(2.0)_, PHMoD_(1.5)_, PHMoD_(1.0)_, PHMoD_(0.5)_ and PHMoD_(0)_ with or without NIR.

Gram-positive *S.aureus* and gram-negative *E.coli* were selected as representative bacterial species for evaluating the antibacterial performance of the PHMoD. The bacterial survival was analyzed via the spread plate method after treatment with the PHMoD. Significant differences in the bactericidal efficiency against *S.aureus* among the PHMoD with different MoS_2_ concentrations existed. The survival rates of *S.aureus* after treatment with PHMoD_(2.0)_, PHMoD_(1.5)_, PHMoD_(1.0)_, PHMoD_(0.5)_ and PHMoD_(0)_ under NIR irradiation were 0.24%, 17.22%, 21.77%, 50.71% and 97.63%. Meanwhile, the survival rate of *S.aureus* without NIR irradiation was 62.73%, 71.21%, 68.79%, 78.79% and 97.52%. The results indicated that PHMoD_(2.0)_ effectively killed *S.aureus* via PTT (Figure 3A and B). The NIR irradiation of the hydrogel can produce local heat and lead to the destruction of the structural integrity of the bacteria, which in turn causes the leakage of genetic material and death. Similarly, PHMoD_(2.0)_, PHMoD_(1.5)_ and PHMoD_(1.0)_ had significant bactericidal effects on *E.coli* under NIR treatment, with PHMoD_(2.0)_ killing approximately 100% of *E.coli*.

The survival rates of *E.coli* after treatment with PHMoD_(2.0)_, PHMoD_(1.5)_, PHMoD_(1.0)_, PHMoD_(0.5)_ and PHMoD_(0)_ under NIR irradiation were 0.58%, 13.57%,15.5%,34.88% and 95.02%, respectively (Figure 3C and D). The survival rate of *E.coli* without NIR irradiation was 55.42%, 62.79%, 72.86%, 91.47% and 90.06%, respectively. The different survival rates suggest that the MoS_2_ concentration in the PHMoD plays a crucial role in its bactericidal effect. These findings indicate that PHMoD_(2.0)_ + NIR has a remarkable bactericidal effect on gram-positive and gram-negative bacteria. When the environmental temperature reaches 50°C and above, the bacteria can be killed quickly by destroying their intracellular protein and enzyme structure [45]. The PHMoD_(2.0)_ temperature reaches 63.2°C after 15 min of NIR, which meets the required temperature for sterilization. Therefore, the PHMoD is suitable for wound environments susceptible to bacterial infections.

Next, the morphologies of *S.aureus* and *E.coli* after treating them with different MoS_2_ of PHMoD and with/without NIR irradiation. SEM images reveal surface crumpling and rupture with dead bacteria. The NIR irradiation of the surfaces of *S.aureus* treated with PHMoD_(2.0)_, PHMoD_(1.5)_ and PHMoD_(1.0)_ resulted in significant wrinkling (Figure 3E). This suggests that PHMoD_(2.0)_, PHMoD_(1.5)_ and PHMoD_(1.0)_ had a strong bactericidal effect on *S.aureus*. Significant damage to the edges of *E.coli* was observed after treatment with PHMoD under NIR (Figure 3F). The NIR irradiation severely damaged the structures of *S.aureus* and *E.coli*, resulting in bacterial death.

### Antioxidant properties of composite hydrogel

**Figure 4.**
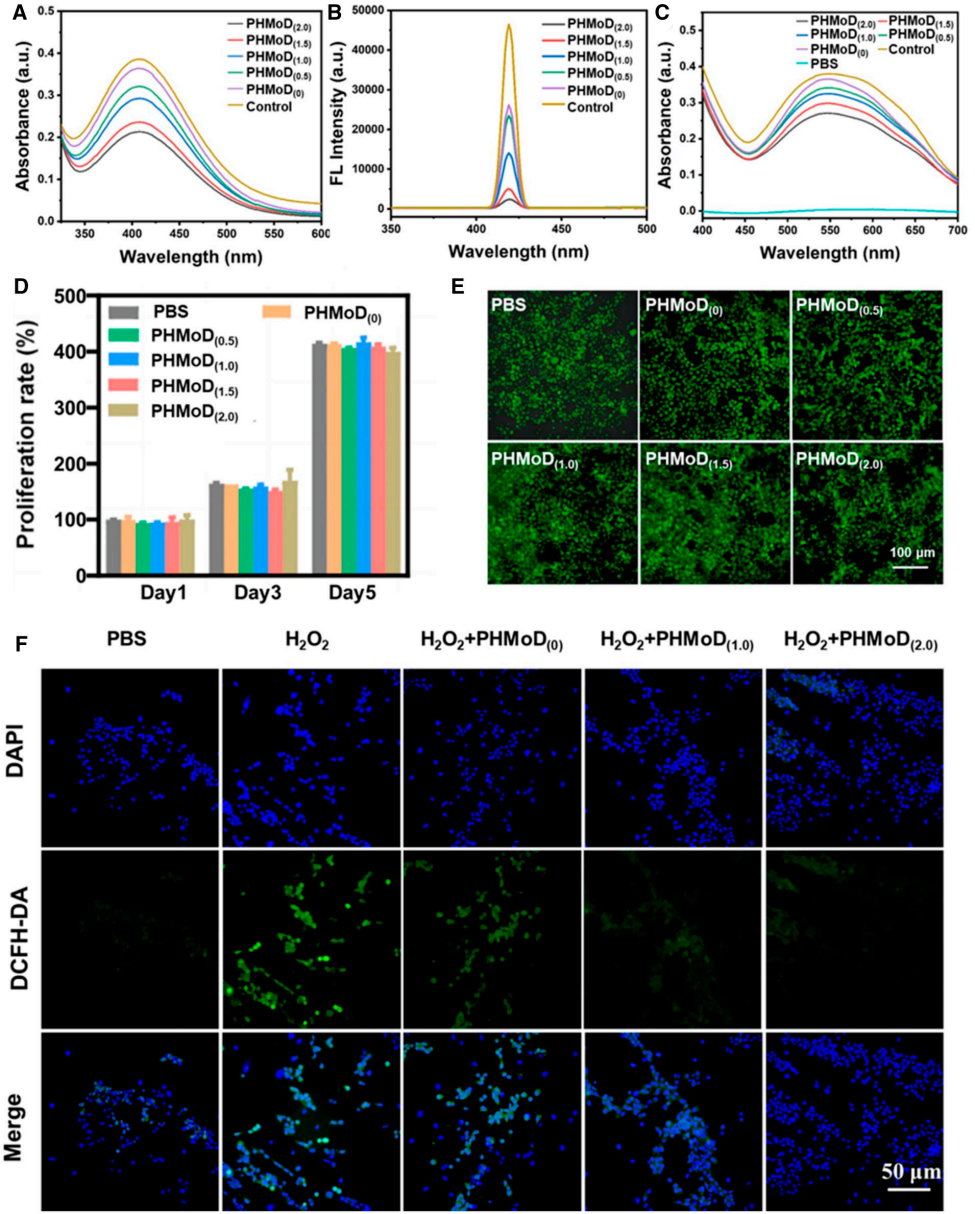
Antioxidant properties of the PHMoD. (**A**) UV–vis spectra of H_2_O_2_ consumption of the hydrogel. (**B**) Fluorescence spectra of •OH scavenging. (**C**) UV–vis spectra of O_2_•^−^ consumption with hydrogel treatment. (**D**) Cell proliferation for L929 cells co-cultured with PHMoD. (**E**) Dead/live cell assay of L929 cells after co-culturing with the PHMoD for 24 h. (**F**) Fluorescence images of L929 cells under various treatments with the PHMoD.

The PHMoD exhibited antioxidant properties, scavenging ROS, including H_2_O_2_, •OH and O_2_•^−^. The simulated peroxidase activity of the hydrogel was investigated using a colorimetric method with titanium sulfate. The UV–visible (UV–vis) spectra of the PHMoD after the addition of H_2_O_2_ exhibited a gradual decrease in the absorption peak intensity at 415 nm as the nanozyme content in the hydrogel increased (Figure 4A). PHMoD_(2.0)_ had the minor absorption peak, while PHMoD_(0)_ exhibited a slight decrease compared with the control. These results indicated that PHMoD_(2.0)_ had an excellent H_2_O_2_ scavenging ability. Next, the scavenging capacity of PHMoD for •OH using terephthalic acid was evaluated. Among ROS, •OH is the most reactive free radical and can cause significant damage to cells. The scavenging rate of •OH increased with the nanozyme content in the PHMoD, and it was demonstrated that PHMoD_(2.0)_ could scavenge •OH (Figure 4B). The scavenging ability of the PHMoD on superoxide radicals was evaluated according to inhibit the photochemical reduction of NBT in the NADH-NBT-PMS system. The absorption spectrum of PHMoD_(2.0)_ exhibited a reduced peak intensity near 560 nm, confirming that PHMoD_(2.0)_ effectively scavenged O_2_•^−^ (Figure 4C). As the concentration of the nanozyme in the PHMoD increased, the O_2_•^−^ the scavenging effect was enhanced. In conclusion, the experimental results suggest that PHMoD can significantly reduce oxidative damage, maintain redox balance, prevent excessive ROS production and reduce the inflammatory response.

After the PHMoD with different MoS_2_ concentrations was co-cultured with L929 cells for 24 h, the cell viability was close to 100% (Supplementary Figure S7A). Cell viability tests after 3 and 5 days indicated no cytotoxicity when the PHMoD treatment time was increased (Supplementary Figure S7B and C). *In vitro*, cell proliferation assays were performed with L929 cells co-cultured with hydrogel. The results indicated that L929 cells incubated with the PHMoD proliferated continuously over a 5-day incubation period (Figure 4D). Live/dead staining tests were performed after coincubation of the PHMoD with cells. The results indicated almost no cell death, confirming that the PHMoD was almost non-cytotoxic (Figure 4E).

In addition, PHMoD_(0)_, PHMoD_(1.0)_ and PHMoD_(2.0)_ hydrogel were investigated for intracellular ROS scavenging in L929 cells in a pathological oxidative microenvironment. After incubation with 100 µM H_2_O_2_ and materials in the L929 cell medium for 24 h, the intracellular ROS production was characterized using the ROS indicator 2,7-dichlorodihydrofluorescein diacetate (DCFH-DA) and photographed under a fluorescence microscope. The intracellular ROS content for the PHMoD group was significantly lower than that for the H_2_O_2_ group, indicating that the PHMoD has a practical antioxidant effect (Figure 4F). Meanwhile, PHMoD_(2.0)_ was the most effective hydrogel for intracellular ROS scavenging in L929 cells in the pathological oxidative microenvironment.

### Wound-healing ability of composite hydrogel

**Figure 5.**
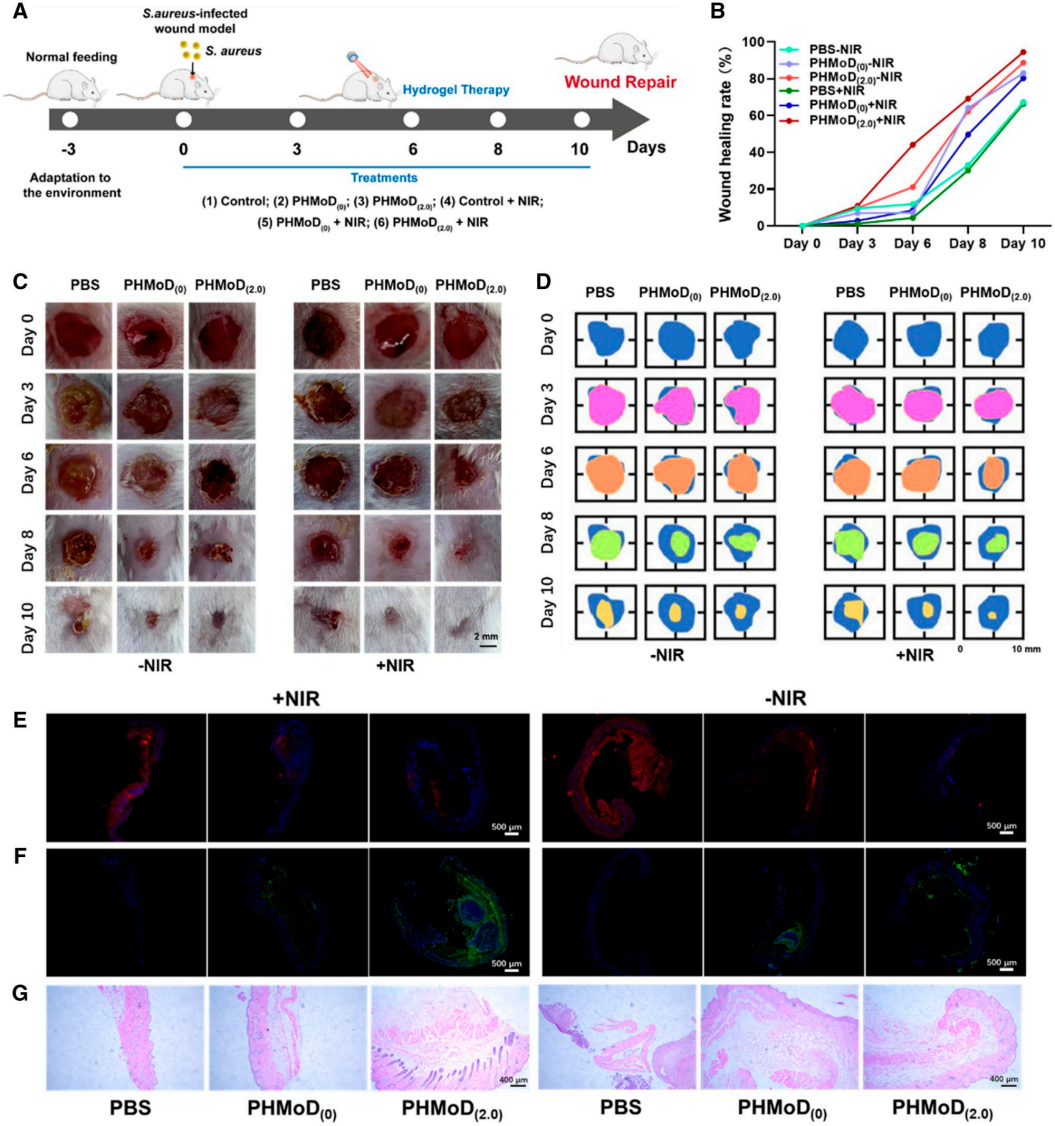
Antimicrobial activity of the PHMoD in an *in vivo* bacterial infection wound model. (**A**) Operation process of PHMoD treatment for the bacterial infection wound model. (**B**) Wound healing rate. (**C**) Images of the wound subjected to each treatment. (**D**) Traces of wound closure over 10 days with different treatments. Immunofluorescence staining of (**E**) IL-6 and (**F**) Ki-67 in the wound bed at 10 days. (**G**) Histomorphological assay of wounds subjected to different treatments at 10 days.

A mouse wound bacterial infection model was established by completely excising the skin at the wound site, leaving only subcutaneous tissue. Six experimental groups were included (Control, PHMoD_(0)_, PHMoD_(2.0)_, Control + NIR, PHMoD_(0)_ + NIR and PHMoD_(2.0)_ + NIR), and the wound-healing process was recorded (Figure 5A). In the NIR group, laser radiation (1.5 W/cm^2^) was used to warm the wounds to 50°C, followed by continuous irradiation for 5 min while ensuring that the real-time temperature did not exceed 55°C using a thermal imager, avoiding burning the skin tissue of the mice. The results indicated significant differences in the wound-healing process among the different groups. The PHMoD_(2.0)_ + NIR group had the best antimicrobial effect, with no visible signs of infection (Figure 5C). For this group, most wounds formed scabs by day 3. In contrast, for the other groups, the wounds appeared infected by day 3, and they improved until day 6, possibly owing to the gradual recovery of the immune system. The wound-healing trajectory over 10 days indicated that for the hydrogel treatment group, the wounds were mostly closed, with smooth skin and good healing (Figure 5D). Plot of wound healing rates are shown in Figure 5B. Remarkably, the wound recovery speed registered on day 10 for the cohort administered with PHMoD_(2.0)_ + NIR (94.47%) markedly surpassed that of the control set treated with PBS+NIR (66.30%) and PBS alone (67.25%), underscoring the outstanding effectiveness of PHMoD_(2.0)_ in fostering wound mending. In contrast, for the control group, the wounds were not yet completely healed and still had small amounts of scabbing. The results indicated that PHMoD_(2.0)_ significantly accelerated wound healing, and PHMoD_(2.0)_ + NIR had the best wound-healing promotion effect. This is attributed to the hydrogel has excellent hygroscopicity to absorb fluid exuded from the wound and a high-water content that provides a moist healing environment for the wound. The incorporation of MoS_2_ nanosheets within PHMoD demonstrates exceptional photothermal conversion efficiency, rendering them promising for NIR-induced PTT targeted at bacterial eradication. Upon achieving temperatures surpassing 50°C, the resultant elevated temperature effectively disrupts intracellular proteins and enzyme structures, leading to rapid bactericidal effects. Furthermore, the inherent antioxidant properties arising from the catalytic activity of MoS_2_, particularly in catalyzing catalase, exhibit significant potential in addressing infections at wound sites. This catalytic activity contributes to the reduction of ROS levels, thereby mitigating oxidative stress and fostering enhanced wound healing mechanisms. Meanwhile, PHMoD possesses both antimicrobial and antioxidant functions, rendering it suitable for application as a wound dressing, particularly for wounds in specialized areas with frequent activity. PHMoD eliminates ROS from the wound and diminishes their concentration to facilitate regeneration at the injured site. Moreover, the exceptional mechanical properties of PHMoD hydrogel enable rapid deformation in response to changes in the active site, ensuring tight adherence and preventing bacterial invasion, thereby reducing the risk of infection. Combining PHMoD_(2.0)_+NIR and photothermal antibacterial therapy can effectively prevent bacterial infection and promote wound healing.

The levels of the inflammatory factor IL-6, which is secreted by mononuclear macrophages and is a critical component of the immune and inflammatory responses, were assessed at the wound site. The level was significantly lower for the PHMoD_(2.0)_ + NIR group than for the control group (Figure 5E). Ki-67 is a nuclear protein associated with ribosomal RNA transcription used to characterize active cell proliferation. The Ki-67 expression was higher for the PHMoD_(2.0)_ + NIR group than for the control group, suggesting more cell proliferation in the wound (Figure 5F). Then, histological staining assess the effects of PHMoD_(2.0)_ on wound healing with/without NIR irradiation. After 10 days of PHMoD_(2.0)_ + NIR treatment, the basic structure of the epithelium and dermis was formed in the wound, with a uniform distribution of hair follicles. The control group had fewer hair follicles and thinner skin, indicating that the wound repair was better with PHMoD_(2.0)_ + NIR (Figure 5G).

In addition, hematoxylin and eosin staining was performed on major organs (heart, liver, spleen, lungs and kidneys) of mice after 10 days of treatment to investigate the *in vivo* safety. No significant inflammatory accumulation was detected in the major organs in all the groups, indicating that the material system is highly biocompatible (Supplementary Figure S8). Routine blood analysis was performed on the mice, and all the indicators were within the normal ranges (Supplementary Figure S9). The hydrogel appeared to have no adverse effects on the mice, suggesting their biocompatibility and safety. The hydrogel can fit well into the wound and retain significant water. Additionally, the PHMoD has exceptional mechanical properties that allow it to maintain its structural integrity, facilitate gas exchange, and resist external stimuli, creating an optimal environment for wound repair. Moreover, the PHMoD can effectively remove ROS from the wound, reducing inflammation, promoting skin regeneration and reducing the ROS concentration. Thus, it is a promising therapeutic option for wound-healing applications.

## Conclusion

In this study, PHMoD was prepared using a MoS_2_–DTT nanozyme with antibacterial and antioxidant functions for the treatment of infected wounds. It exhibited exceptional physicochemical properties, such as mechanical strength, swelling ability, photothermal properties, cytocompatibility, ROS scavenging capability, antibacterial activity and wound-healing efficacy in animal models. Its antibacterial activity against gram-positive bacteria (*S.aureus*) and gram-negative bacteria (*E.coli*) was assessed, and it achieved bactericidal rates of 99.76% and 99.42%, respectively, reducing the risk of wound infection and facilitating wound healing. Furthermore, increasing the content of MoS_2_–DTT nanozyme in the PHMoD reduced the levels of H_2_O_2_, •OH, O_2_•^−^ and intracellular ROS, indicating that the PHMoD effectively scavenged ROS and functioned well as an antioxidant. In models of infected wounds, the PHMoD was effective for promoting wound healing, achieving complete wound closure and smoothing the skin within 10 days. Therefore, the MoS_2_–DTT nanozyme-based PHMoD is useful for treating infected wounds and has many potential applications.

## Supplementary Material

rbae025_Supplementary_Data

## Data Availability

All data analyzed during this study are included in this published article (Supplementary data). Other raw data required to reproduce these findings are available from the corresponding author on reasonable request.
