# Supplementary material for: Antibacterial and antioxidant bifunctional hydrogel based on hyaluronic acid complex MoS2–dithiothreitol nanozyme for treatment of infected wounds
Source: Regen Biomater. 2024 Mar 9;11:rbae025. doi: 10.1093/rb/rbae025 (PMC11009022; doi:10.1093/rb/rbae025)
Supplement: rbae025_Supplementary_Data [file rbae025_supplementary_data.docx]

Supporting Information

**Antibacterial and antioxidant bifunctional hydrogel based on hyaluronic acid complex MoS_2_-dithiothreitol nanozyme for treatment of infected wounds**

*Yongping Lu ^1,3, †^, Weiqi Kang ^1, †^, Yue Yu ^1, †^,* *Ling Liang ^1^, Jinrong Li ^1^, Haiying Lu ^1^, Ping Shi ^1^, Mingfang He* *^1^, Yuemin Wang ^2, 3,^ *, Jianshu Li ^3^, and Xingyu Chen ^2,^ **

a Guangyuan Central Hospital, Guangyuan 628000, P. R. China.

b College of Medicine, Southwest Jiaotong University, 610003, China

c College of Polymer Science and Engineering, State Key Laboratory of Polymer Materials Engineering, Sichuan University, Chengdu 610065, P. R. China.

* Corresponding authors at: College of Medicine, Southwest Jiaotong University, 610003, China

E-mail addresses: wym291313480@163.com (Y. Wang), chenxy@swjtu.edu.cn (X. Chen).

‡ These authors contributed equally.

# 1.Experimental Section

## *1.1. Chemicals*

Molybdenum disulfide and hyaluronic acid (80–150 WDa) were purchased from Shanghai Yuanye Biotechnology Co., Ltd. (Shanghai, China). Dithiothreitol, water-soluble diphenyl(2,4,6-trimethylbenzoyl) phosphine oxide (TPO)-based nanoparticle photoinitiator, p-phthalic acid, paraformaldehyde, a hydrogen peroxide solution, NBT, sulfuric acid, and titanium sulfate were purchased from Aladdin Biochemical Technology Co., Ltd. (Shanghai, China). Pluronic F127, NADH, and PMS were purchased from Sigma–Aldrich (Shanghai, China). Methacrylic anhydride, glutaraldehyde, ethanol anhydrous, and acryloyl chloride were purchased from Shanghai Macklin Biochemical Technology Co., Ltd. (Shanghai, China). Triethylamine was purchased from Tianjin Chemio Reagent Co., Ltd. (Tianjin, China). Chloroform, calcium chloride, sodium chloride, dichloromethane, and sodium hydroxide were purchased from Chengdu Cologne Chemical Co., Ltd. (Chengdu, China). Cellulose dialysis membranes (3500 and 14000 Da) were purchased from Xi’an Yobios Biotechnology Co., Ltd. (Xi’an, China). Tris and Tris-HCl were purchased from BioFroxx (Einhausen, Germany). High-glucose cell medium (Dulbecco’s Modified Eagle Medium), PBS, BSA, fetal bovine serum (FBS), penicillin–streptomycin solution, and trypsin were purchased from Chengdu Baoxin Biotechnology Co., Ltd. (Chengdu, China). L929 cells, cell counting kit-8 (CCK-8), fluorescein diacetate, p-phenylenediamine (PDA), 2',7'-dichlorodihydrofluorescein diacetate (DCFH-DA), and propidium iodide (PI) were purchased from MedChemExpress (Princeton, New Jersey, USA). *S. aureus* (ATCC 6538), *E. coli*, nutrient broth medium, and solid agar medium were purchased from Guangdong Huankai Microbial Technology Co., Ltd. (Guangzhou, China). Healthy male kunming rats (30–40 g) were purchased from Chengdu Dossy Experimental Animals Co., Ltd. (Guangzhou, China).

*1.2. Characterization*

To ensure the uniform dispersion of samples, PF127-DA, MoS_2_ nanosheets, MoS_2_-DTT nanozyme, PHMoD were mixed homogeneously via ultrasonication. It was tested via TEM (G2 F20 S-TWIN, Thermo Fisher Scientific, USA), AFM (Smart SPM, AIST-NT, USA), SEM (SU8220, Hitachi, Japan), XPS (K-Alpha Plus, Thermo Fisher Scientific, USA), XRD analysis (Ultima IV XRD diffractometer, Rigaku Corp, Japan), and FTIR spectroscopy (Nicolet iS50, Thermo Fisher Corp, USA). The ^1^H NMR spectra of PF127-DA were acquired using a Bruker AV III HD 400-MHz instrument (Nikon Corp, Japan) with deuterated chloroform as the solvent. The static water contact angles of PHMoD_(2.0)_ and PHMoD_(0)_ were measured using a DSA25 optical contact angle meter (Kruss, Germany). Three-phase boundary images of the water-droplet contour lines were captured using a charge-coupled device camera and were numerically fitted and calculated.

*1.3. Mechanical properties*

The rheological properties of the PHMoD hydrogel (diameters of 10 mm and thicknesses of 2 mm) were tested using a modular intelligent advanced rotational rheometer (MCR302, Anton Paar, Austria) fitted with a standard stainless-steel conical plate (diameter of 8 mm). Dynamic frequency sweep tests were performed on PHMoD_(0)_ and PHMoD_(2.0)_ gel at a fixed shear strain of 1% at room temperature in the frequency range of 0.01–10 Hz. Dynamic amplitude sweep tests were performed at a fixed oscillation frequency of 1 Hz with a strain range of 1%–100%, and the changes in the energy storage modulus (G′) and loss modulus (G") of the PHMoD gel were recorded.

*1.4. Cytocompatibility*

The cell activity was assessed using Cell Counting Kit-8 (CCK-8), and PDA/PI (p-phenylenediamine/propidium iodide) dye was used for cell staining. To investigate cell viability and proliferation, L929 cells were inoculated in 48-well plates at a density of 1.5 × 10^4^ cells per well and incubated for 24 h. PHMoD hydrogel (8 mm in diameter and 2 mm in thickness) was sterilized via immersion in 75% ethanol for 24 h. After sterilization, the hydrogel was immersed in PBS and changed every hour. This process was performed five times. Finally, we co-incubated the hydrogels with cells for 24, 48, and 72 h and quantified cell viability using the CCK-8 kit.

To perform cell death staining, L929 cells were inoculated in 48-well plates at a density of 1.5 × 10^4^ cells per well and incubated for 24 h. After co-culturing the cells with sterilized PHMoD hydrogel for 24 h, the cells were stained using a live/dead staining kit. Next, the cells were observed and recorded using a fluorescence microscope. The round full-thickness cutaneous wounds were created on the necks of mice to study the effectiveness of the PHMoD for wound repair.

*1.5. Hyaluronidase stability of PHMoD*

The hydrogel was soaked in PBS for 24 h and weighed them (M_0_). Next, 3 mL PBS solution containing hyaluronidase (100 U/mL) was added, and the resulting mixture was placed on a shaker at 37 °C. At fixed intervals, the hydrogel (M_t_) was weighed, and the degradation rate was calculated as follows:

$$Degradation rate=\frac{M_{t}}{M_{0}}\times100\%$$

where M_t_ represents the weight of the hydrogel at a fixed time point, and M_0_ represents the initial weight of the hydrogel.

*1.6. Histological analyses*

All experimental mice were treated with hydrogel after 10 days. Relevant traumatic tissues and major organs (heart, liver, spleen, lung, and kidney) were collected and fixed with 4% paraformaldehyde fixative. The fixed tissues were then dehydrated and paraffin-embedded using an automatic dehydrator. The samples were sectioned and dewaxed, followed by staining with hematoxylin for 10–20 min and rinsing with water for 2 min. The samples are fractionated with hydrochloric acid in alcohol for 5-10 s and then rinsed for 2 min. Next, the sections are placed in warm or weakly alkaline aqueous solution at 50°C until a blue appears, rinsed for 2 min and placed in 85% alcohol for 3-5 min. Sections were stained with eosin for 3-5 min, washed with water, subjected to alcohol gradient dehydration and sealed with xylene clear neutral adhesive and finally examined microscopically for histological analysis.

*1.7. Immunofluorescence analyses*

Fixed tissues were dehydrated by an automatic dehydrator, embedded, and sectioned, and the dewaxed sections were immersed in 0.01 M citrate buffer (pH 6.0). The sections were heated in a microwave oven high. Every 5 min, the above operation was performed once, and then the samples were cooled to room temperature. The samples were washed with PBS for 5 min three times, followed by dropwise addition of a 10% serum blocking solution. And then, it was kept at room temperature for 30 min. Then, the primary antibody (1:100 IL-6 and 1:50 Ki-67) was added dropwise overnight at 4 ℃, followed by 5 min of PBS washing, which was repeated three times. Subsequently, the secondary antibody (1:100 fluorescein isothiocyanate-labeled goat anti-rabbit IgG) was added dropwise at 37 ℃ for 30 min, followed by dropwise addition of DAPI. The samples were then incubated at room temperature for 10 min, followed by PBS washing for 5 min, which was repeated three times. It was sealed with an anti-fluorescence decay blocker.

**
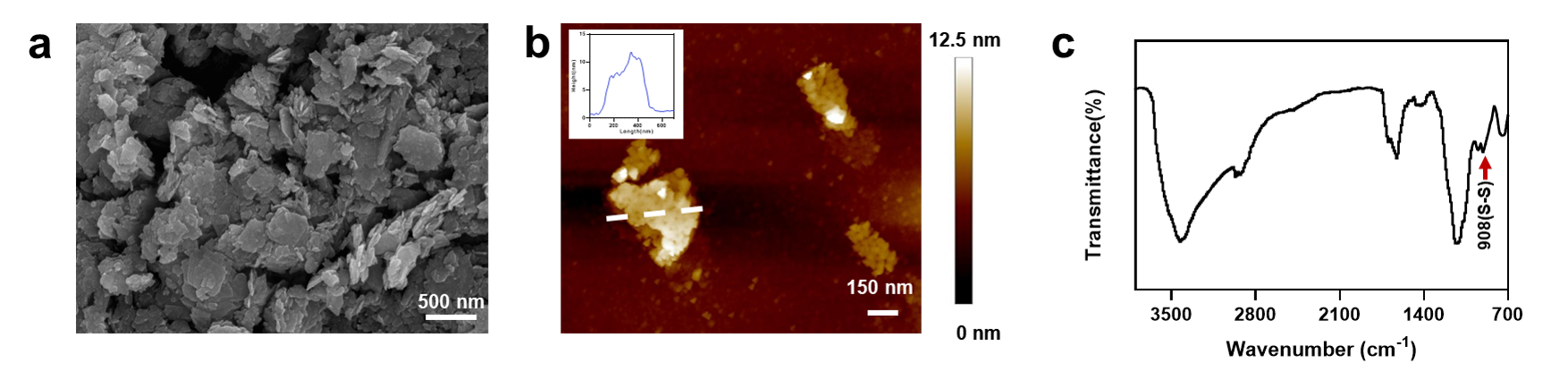
**

**Figure S1.** (a) SEM image of MoS_2_ nanosheet. (b) AFM image and height profile of MoS_2_ nanosheet. (c) FTIR spectrum of MoS_2_ nanosheet.


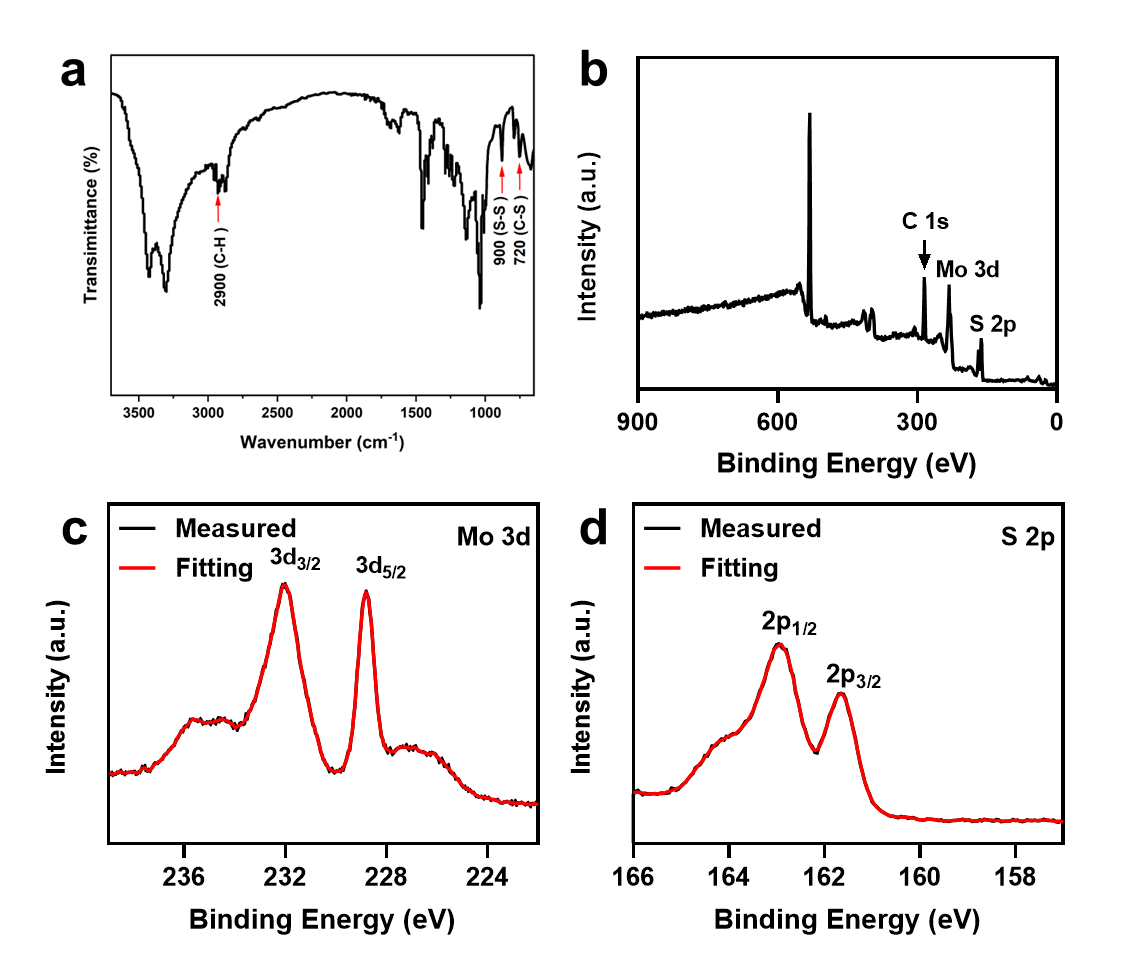


**Figure S2.** (a) FTIR spectrum of MoS_2_-DTT. (b) XPS spectrum of MoS_2_-DTT. (c) Mo 3d XPS spectrum. (d) S 2p XPS spectrum.


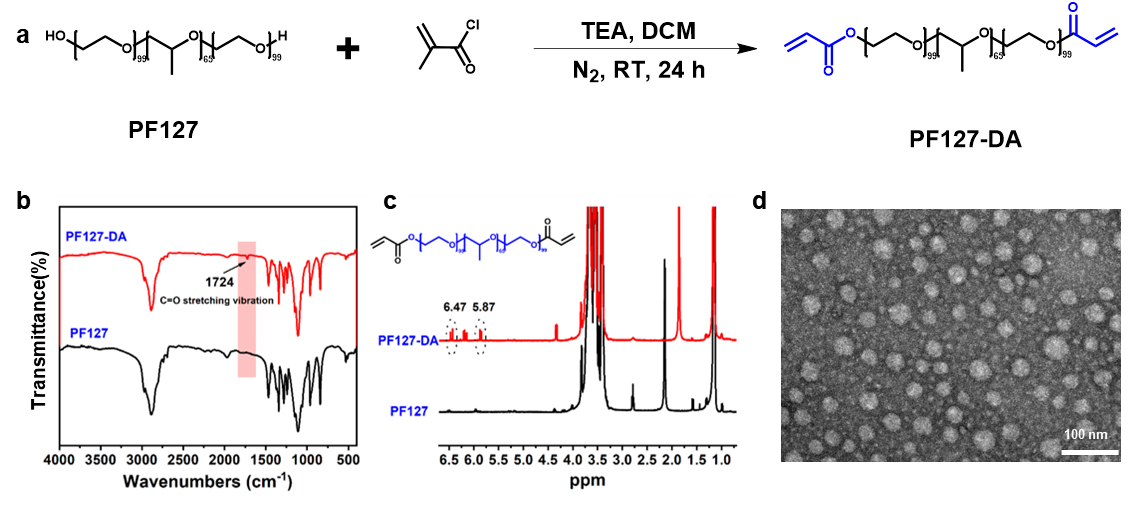


**Figure S3.** (a) Synthesis of PF127-DA. (b) FTIR spectra of PF127 and PF127-DA. (c) ^1^H NMR spectra of PF127 and PF127-DA. (d) TEM image of PF127-DA.


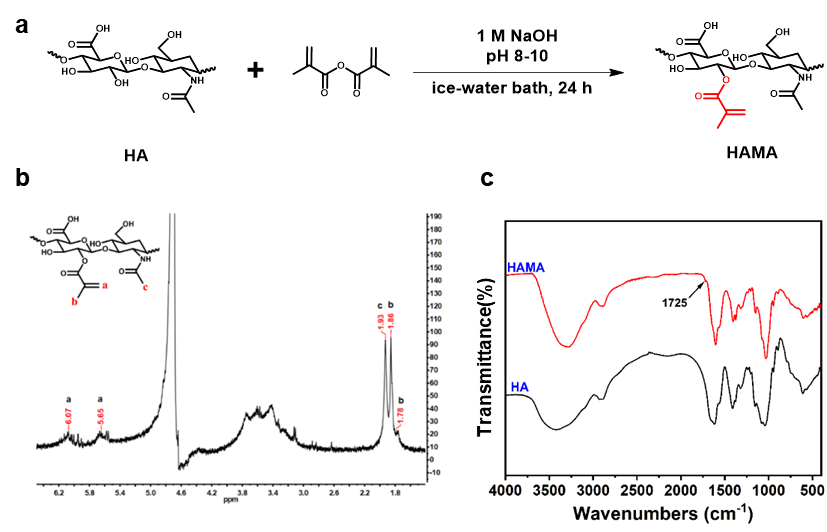


**Figure S4.** (a) Synthesis of HAMA. (b) ^1^H NMR spectrum of HAMA. (c) FTIR spectra of HA and HAMA.


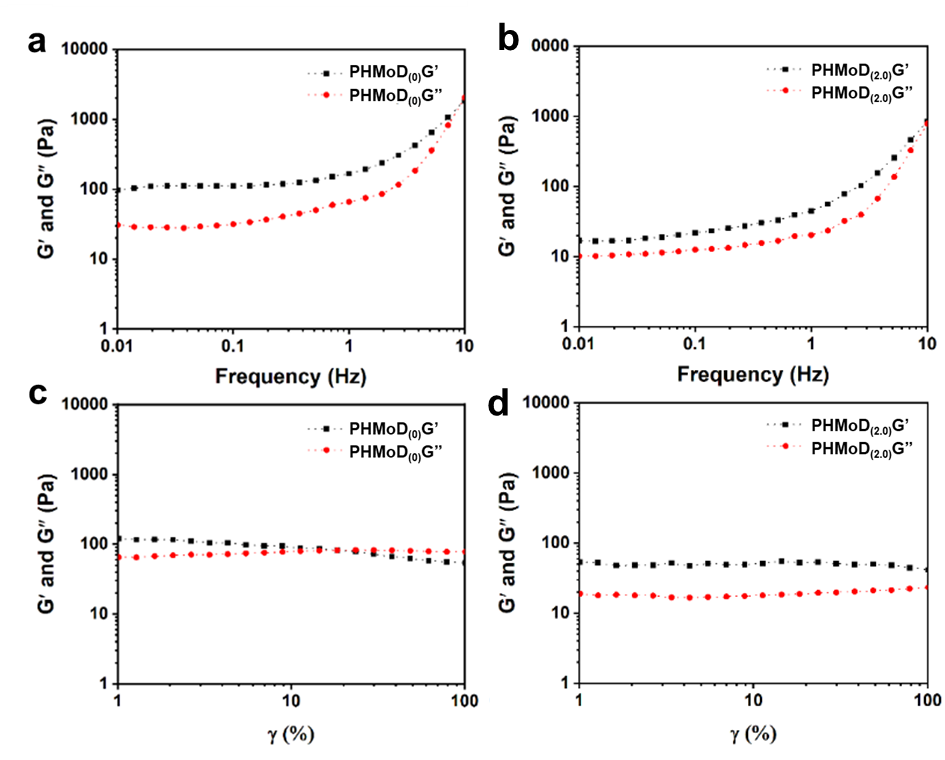


**Figure S5.** Rheological behaviors of PHMoD_(0)_ and PHMoD_(2.0)_ hydrogels. (a) Dynamic frequency sweep of PHMoD_(0)_ hydrogels and (b) PHMoD_(2.0)_ hydrogels from 0.01 to 10 Hz with 1% strain. (c) Dynamic strain sweep of PHMoD_(0)_ hydrogels and (d) PHMoD_(2.0)_ hydrogels from 1% to 100% strain at 1 Hz.

**Figure S6.** Degradation behaviors of PHMoD_(0)_, PHMoD_(0.5)_, PHMoD_(1.0)_, PHMoD_(1.5)_, and PHMoD_(2.0)_.


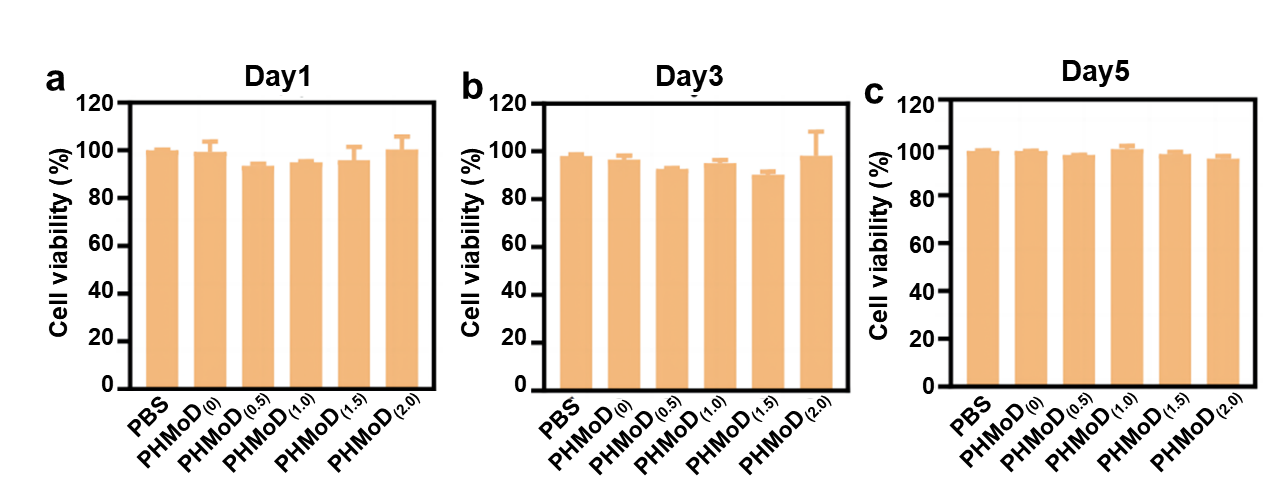


**Figure S7.** Cell viability after (a) 1 d, (b) 3 d, and (c) 5 d of co-culturing L929 cells with the PHMoD.


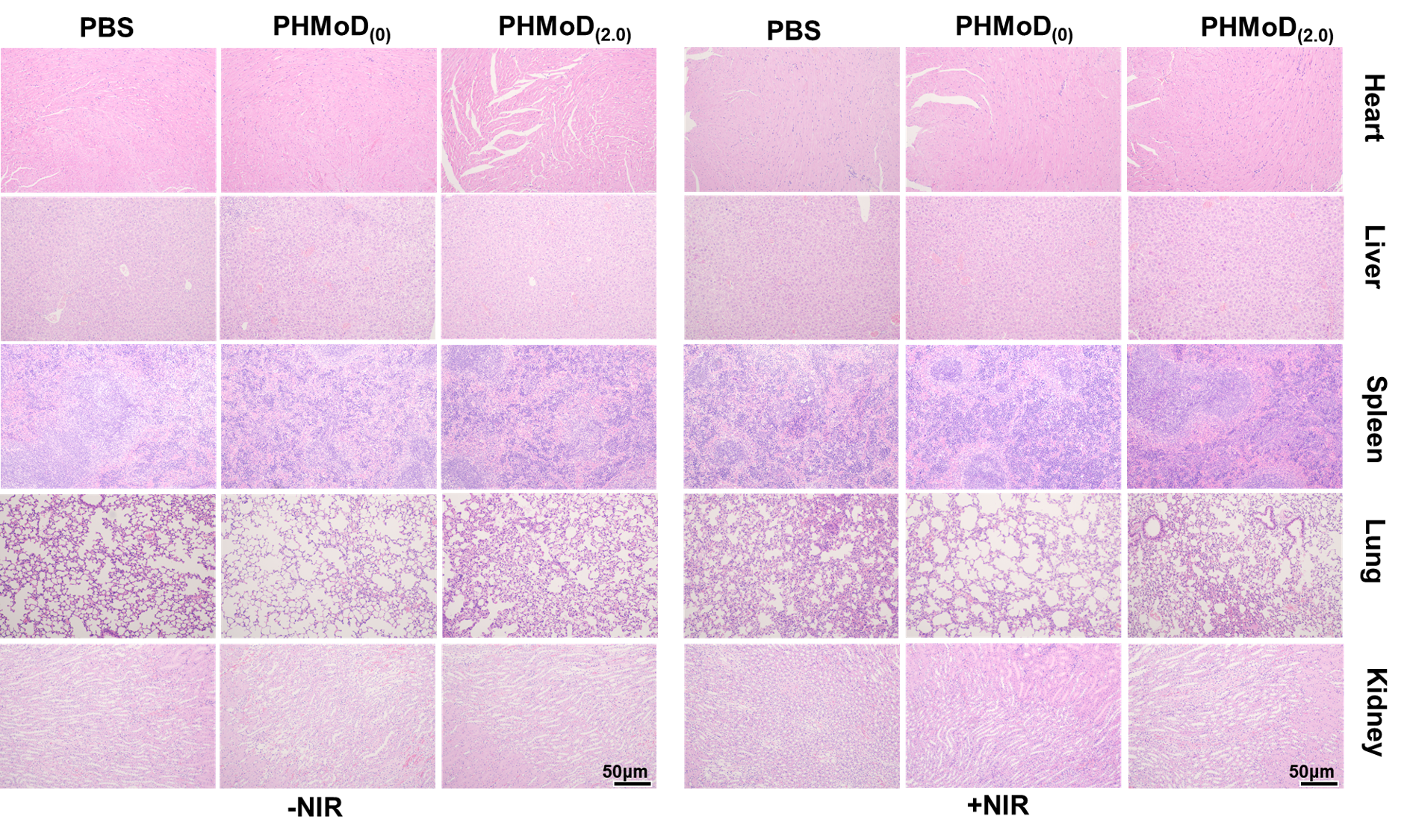


**Figure S8.** H&E staining of major organ tissue slices harvested from mice after 15 d of treatment.


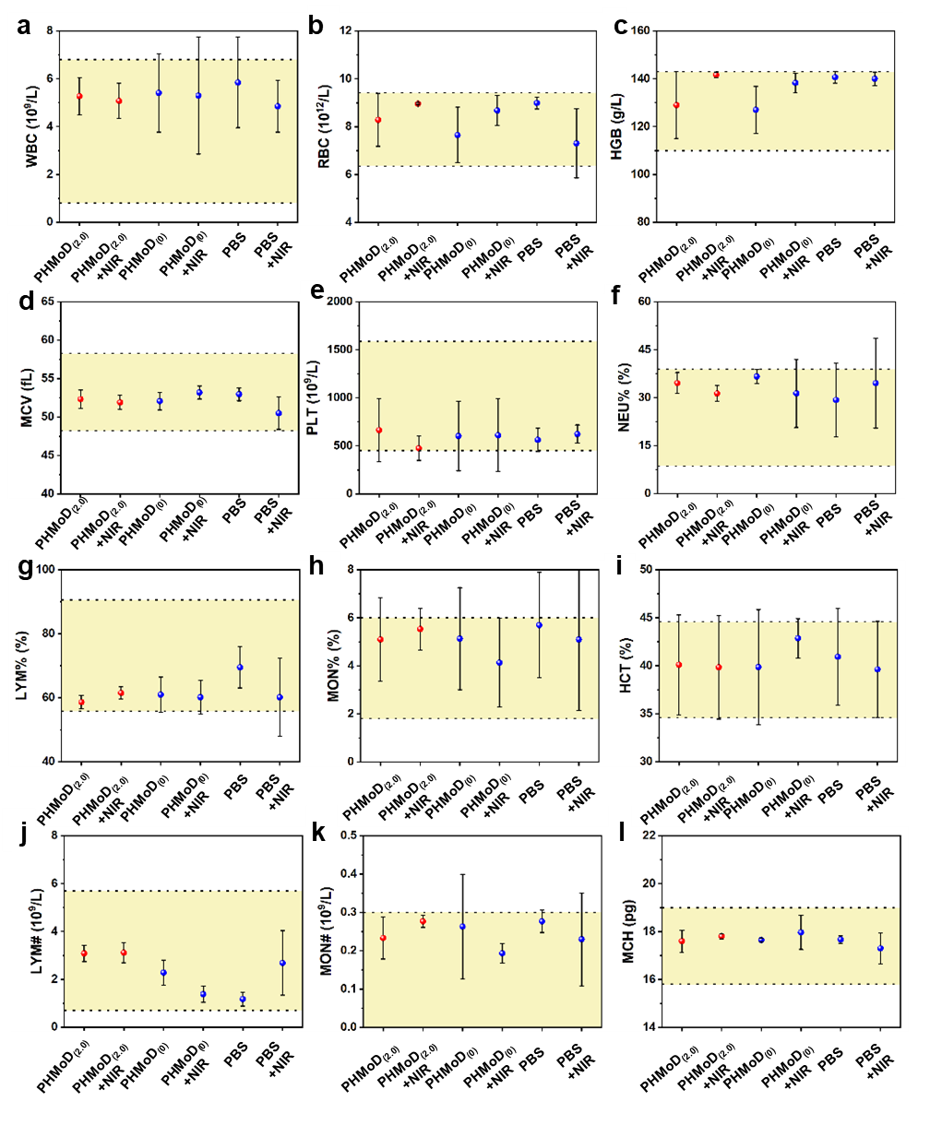


**Figure S9.** Blood routine data of mice treated with PHMoD_(0)_ and PHMoD_(2.0)_ hydrogels and the PBS solution for bacterial neck wounds with/without NIR irradiation.
